# Vitamin D, a Regulator of Androgen Levels, Is Not Correlated to PSA Serum Levels in a Cohort of the Middle Italy Region Participating to a Prostate Cancer Screening Campaign

**DOI:** 10.3390/jcm12051831

**Published:** 2023-02-24

**Authors:** Felice Crocetto, Biagio Barone, Giulio D’Aguanno, Alfonso Falcone, Rosamaria de Vivo, Monica Rienzo, Laura Recchia, Erika Di Zazzo

**Affiliations:** 1 Department of Neurosciences, Reproductive Sciences and Odontostomatology, University of Naples Federico II, 80131 Naples, Italy; 2 Emergency Medicine Unit, Department of Clinical Internal, Anesthesiological and Cardiovascular Sciences, Sapienza University of Rome, 00161 Rome, Italy; 3 Department of Medicine and Health Sciences “V. Tiberio”, University of Molise, 86100 Campobasso, Italy; 4 Department of Environmental, Biological, and Pharmaceutical Sciences and Technologies, University of Campania “Luigi Vanvitelli”, 81100 Caserta, Italy; 5 UOC Laboratorio Analisi, Ospedale “A. Cardarelli”, 86100 Campobasso, Italy

**Keywords:** prostate cancer, lifestyle, PSA, vitamin D

## Abstract

Prostate cancer (PCa) is the most common non-cutaneous malignancy in men worldwide, and it represents the fifth leading cause of death. It has long been recognized that dietary habits can impact prostate health and improve the benefits of traditional medical care. The activity of novel agents on prostate health is routinely assessed by measuring changes in serum prostate-specific antigen (PSA) levels. Recent studies hypothesized that vitamin D supplementation reduces circulating androgen levels and PSA secretion, inhibits cell growth of the hormone-sensitive PCa cell lines, counteracts neoangiogenesis and improves apoptosis. However, the results are conflicting and inconsistent. Furthermore, the use of vitamin D in PCa treatments has not achieved consistently positive results to date. In order to assess the existence of a correlation between the PSA and 25(OH)vitamin D levels as widely hypothesized in the literature, we analyzed the serum PSA and 25(OH)vitamin D concentration on a cohort of one hundred patients joining a PCa screening campaign. Additionally, we performed medical and pharmacological anamnesis and analyzed lifestyle, as sport practice and eating habits, by administering a questionnaire on family history. Although several studies suggested a protective role of vitamin D in PCa onset prevention and progression, our preliminary results revealed a clear absence of correlation between the serum vitamin D and PSA concentration levels, suggesting that vitamin D has no impact on PCa risk. Further investigations enrolling a huge number of patients are needed with particular attention to vitamin D supplementation, calcium intake, solar radiation that influences vitamin D metabolism and other potential indicators of health to confirm the absence of correlation observed in our study.

## 1. Introduction

Prostate cancer (PCa) is the third most commonly diagnosed cancer in men, with an estimated 1.4 million diagnoses worldwide in 2021, and it represents the fifth leading cause of cancer death [1,2]. Several risk agents, including genetic factors, elderly, ethnicity, high testosterone levels and lifestyle play a pivotal role in PCa onset [3]. The PCa incidence varies among different geographical areas, showing a high incidence in developed and industrialized countries. The geographical differences are linked to disparities in screening tests frequency and potency among countries with a different development level [4]. In addition, it has been estimated that PCa incidence augments with the increasing age of the patients: all-age incidence is 31 per 100,000 males, with a lifetime cumulative risk of 3.9% and, more than one in four men over 75 years is affected by PCa [5,6].

Prostate-specific antigen (PSA) is a glycoprotein secreted exclusively by prostate epithelial cells [7]. A PSA blood test represents the first step to evaluate suspicious PCa. Serum PSA testing is an early, comfortable and relatively inexpensive marker. However, since PSA is produced by both benign and malignant prostate epithelial cells, this serum marker shows limitations as a screening test for PCa. An elevated serum PSA level, indeed, can be detected in several not-malignant conditions, including prostatitis and benign prostatic hyperplasia (BPH) [8]. Furthermore, the PSA cut-off level is still not standardized, and despite its role as a PCa independent predictor, its use alone could be misleading, conducting to unnecessary biopsies [9,10,11,12]. The low PSA serum level specificity prompted the evaluation of additional markers of PCa risk. In this scenario, several years ago, researchers proposed the use of PSA velocity, considering that men with PCa show a more rapid rate of increase in PSA levels than those without PCa [13]. Recently, liquid biopsy has been proposed as a novel tool for cancer diagnosis and follow-up. It could be speculated that in the next few years, PCa diagnosis could be revolutionized by integrating novel, accurate and specific diagnostic markers with PSA serum level [14,15].

In recent years, a great enthusiasm on the potential vitamin D role in cancer prevention has been registered. It has been hypothesized that high vitamin D levels could counteract indolent PCa progression, considering that African-Americans show low vitamin D levels and high risk of advanced PCa. However, even if racial disparities among vitamin D and PSA levels were reported in the literature, the topic is still controversial, with unclear results in intervention studies [16,17]. Vitamin D bioavailability depends not only on diet and supplement use but also skin biosynthesis in response to ultraviolet B radiation exposure. Accordingly, countries with varying sunlight exposure show a high PCa incidence, whereas increased sun exposure has been suggested to decrease the advanced PCa risk [18,19,20]. In addition to the well-established vitamin D role in calcium homeostasis, it has been demonstrated that vitamin D exerts anti-cancer effects, counteracting inflammation and angiogenesis, and promoting apoptosis [21,22]. PCa tissue and cell lines express vitamin D receptors, while calcitriol exerts anti-proliferative effects in normal prostate epithelial cells [23]. Furthermore, PCa patients showed lower serum vitamin D levels than matched controls, and PCa risk decreased with increasing serum calcitriol levels [24]. In biopsy-naïve men, low levels of both plasma and serum vitamin D are associated with an increased PCa risk compared to high vitamin D levels [25,26].

Additionally, a meta-analysis revealed that an increase of 20 nmol/L plasma vitamin D decreased the overall and PCa specific mortality [27].

Several mechanisms have been proposed to support the putative protective role of vitamin D on prostate health. Vitamin D binds to the vitamin D receptors (VDR), and it is a member of the steroid receptors’ superfamily. Upon vitamin D treatment, a heterodimer VDR-retinoid-X receptors exists. The activated VDR then binds to the promoter region of specific genes with vitamin D response elements (VDREs) to regulate the transcription of genes involved in prostate cell differentiation and metabolism [28,29,30]. Again, the active vitamin D metabolite inhibits the local conversion of dehydroepiandrosterone to active androgens, showing prostate growth-stimulation [31]. However, the literature data show conflicting results, and the topic still remains highly debated. A meta-analysis showed that men with elevated vitamin D serum levels had a higher risk of developing PCa than men with low serum levels of vitamin D [32]. In addition, it seems that the vitamin D serum level could be associated to PCa aggressiveness [25]. Furthermore, some studies suggested a positive association between PSA and high vitamin D serum level in PCa [33].

In addition, an inverse association between solar UV exposure and serum PSA concentration, especially during seasons of low UV (i.e., winter and spring), has been noted [33]. The relationship between vitamin D, UV exposure and serum PSA did not seem to have implications in PCa primary prevention, but some vitamin D supplementation trials have shown that vitamin D supplementation for PCa patients in active surveillance reduced the number of the positive core at the control repeat biopsy and post-radical prostatectomy PSA levels [34,35]. 

Based on the aforementioned literature data, revealing some controversial evidence, and considering the proposal of vitamin D supplementation to prevent PCa incidence, we aim to assess the correlations eventually existing, between serum vitamin D and serum PSA levels in a middle Italy region cohort of men participating in a PCa screening campaign.

## 2. Materials and Methods

### 2.1. Study Sample

All participants are Caucasians living in Molise, a small region in the middle of Italy, attending a prostate cancer screening campaign prompted by Asrem—Azienda Sanitaria Regionale del Molise.

All participants aged between 50 and 70 years were recruited between November 2021 and December 2021 and were asked to complete a questionnaire containing questions about demographic characteristics, prostate health, food, alcohol and smoking habits, drugs and supplements used, medical history and type, duration, and frequency of physical activity, prior to their first clinic visit.

We selected one hundred participants aged between 50 and 70 years “free of prostate disease’ (participants have not previously received a diagnosis of PCa, benign prostatic hyperplasia, or prostatitis and have not referred symptoms suggestive of the aforementioned diseases). Additionally, the patients have been screened with a digital rectal examination that have not indicated a significant increase in gland volume [36]. Digital rectal examination has not affected the inclusion or exclusion criteria. 

We excluded participants under 50 years of age and over 70 years of age with a PCa diagnosis. Participants provided written approved consent. 

### 2.2. Study Design

A retrospective study was conducted utilizing questionnaire data and blood samples which were collected from participants to the “Novembre azzurro” initiative prompted by ASReM (Azienda Sanitaria Regionale del Molise). The retrospective use of data collected for the present study was approved by ASReM (Azienda Sanitaria Regionale del Molise), according to the institution ethical guidelines.

### 2.3. Sample Collection

Fasting blood samples were collected from participants before digital rectal exploration. Whole blood samples were allowed to clot and then centrifugated to separate serum. Serum aliquots were stored at −80 °C until samples were processed. Total PSA and vitamin D analyses were carried out at “Cardarelli” Hospital, Campobasso, Italy, using the Atellica Solution (Siemens Healthineers) analyzer with the Siemens Atellica IM PSA method (Siemens) calibrated against the WHO standard. The Atellica IM PSA method is a 2-site sandwich chemiluminometric immunoassay using constant amounts of 2 antibodies. The first antibody is a goat polyclonal anti-PSA antibody labeled with acridinium ester. The second antibody, contained in the solid phase, is a mouse monoclonal anti-PSA antibody covalently linked to paramagnetic particles.

Serum vitamin D levels were measured on the Atellica Solution (Siemens Healthineers) instrument using the Siemens Atellica IM VitD method (Siemens) calibrated against the WHO standard. The Atellica IM VitD method is a competitive immunoassay using a fluorescein labeled-mouse monoclonal antibody covalently bound to paramagnetic particles, an ester-acridinium labeled anti-25(OH)vitamin D mouse monoclonal antibody, and a fluorescein-labeled vitamin D analogue. The analytical performance has been assessed by control sample (Biorad) measurement that showed values falling within the recommended limits.

Sera were stored frozen at −80 °C until the end of sample collection, after which remaining analyses were performed simultaneously, in duplicate. From the Laboratory Informatics System, we retrieved records that included the following fields: anonymous patient identification number; gender; age; date of measurement; the name of the measured parameter; test results; reference range and unit; and instrument used for testing. The value of 4 ng/mL has been chosen as the cut-off for PSA. Accordingly, men have been divided into two groups based on their PSA serum level: men with a serum PSA levels below 4 ng/mL (normal PSA) and men with serum PSA > 4 ng/mL (abnormal PSA). Vitamin D serum levels were grouped as follows: vitamin D serum concentration < 21 ng/mL (vitamin D deficiency); vitamin D serum concentration between 21 and 30 ng/mL (vitamin D insufficiency); vitamin D serum concentration > 30 ng/mL (vitamin D recommended), values > 100 ng/mL (vitamin D toxicity).

### 2.4. Statistical Analysis

Patients were enrolled using a random sampling technique. Descriptive characteristics of patients involved were expressed as means and standard deviations for continuous variables, while absolute counts and percentages were used for categorical variables. The normality of variable distributions was assessed via the Kolmogorov–Smirnov test. The Chi-square test was used to assess the relationship between PSA and vitamin D expressed as categorical variables (normal versus abnormal PSA for PSA serum levels; deficiency versus insufficiency versus recommended for vitamin D serum levels) in a contingency table while the Spearman’s rank correlation test was used to assess the correlation among PSA and vitamin D expressed as continuous variables. All statistical analyses were conducted using IBM SPSS software (version 27, IBM Corp, Armonk, NY, USA), considering *p*-value < 0.05 as statistically significant.

## 3. Results

A total of one hundred and twenty-five patients were enrolled. The descriptive characteristics of patients involved, obtained from the questionnaire previously mentioned, are reported in Table 1. Mean age was 61.14 ± 5.66, while mean Body Mass Index (BMI), obtained from height and weight data, was 27.97 ± 3.53. In addition, 23% of patients regularly take supplements and drugs, reporting, among the most commonly used drugs, antihypertensive, hypolipidemic, hypoglycemic, diuretic, anticoagulant and antiplatelet, hypouricemic, gastroprotective and anti-inflammatory drugs. Overall, 20.6% refer to some kind of disease, with 14.3% and 4.8% reporting hypertension and diabetes, respectively. In addition, only 4% of patients report familiarity for PCa. Regarding smoking habits, 5.6% smoke cigarettes, while 94.4% do not smoke or have quit for over 10 years. Regarding the consumption of alcohol, 18.3% of patients declared to consume a glass of wine or beer with meals. Finally, regarding the sedentary or dynamic lifestyle, 77% of patients reported practicing an hour of brisk walking at least twice a week, while 23% of patients declared a sedentary lifestyle with minimal or no physical activity.

By analyzing PSA concentration, it emerged that 60.3% of patients showed physiological PSA concentration, while 39.7% of patients showed a PSA value higher than the threshold. By analyzing vitamin D concentration, 61.9% of patients showed vitamin D *deficiency* (<21 ng/mL), while 24.6% of patients showed a vitamin D *insufficiency* (values between 21 and 30 ng/mL).

**Figure 1:**
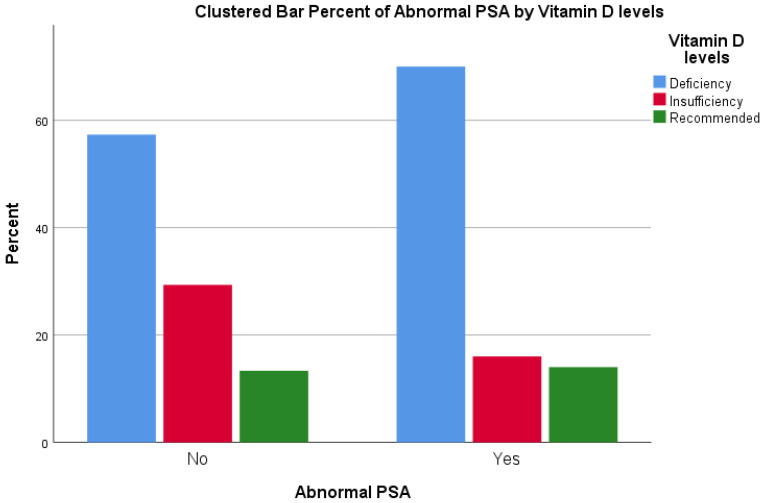
Distribution of vitamin D levels according to normal/abnormal PSA.

Results of the Chi-square test are reported in Table 2 and Figure 1. Overall, 70% of patients with an abnormal PSA reported a vitamin D deficiency compared to 57.3% of patients with a normal PSA. Similarly, 16% and 14% of patients with an abnormal PSA reported, respectively, an insufficiency and a recommended vitamin D level, compared to 29.3% and 13.3% of patients with a normal PSA. Nevertheless, the test did not report a statistical significance, with *p* = 0.223.

**Figure 2:**
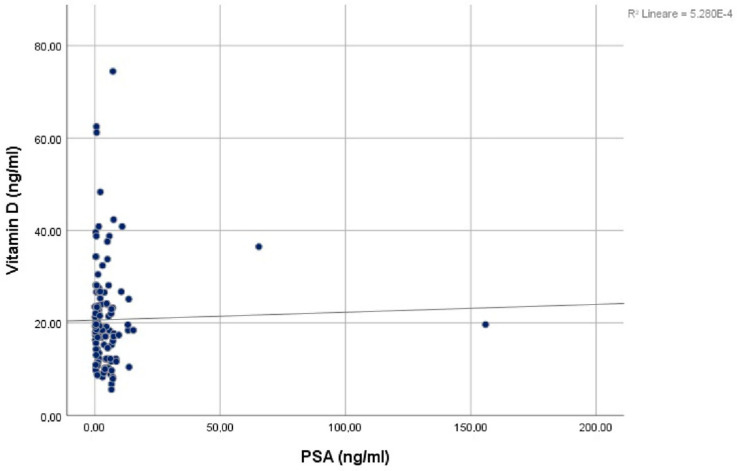
Graphical result of the Spearman’s rank correlation test.

A similar result was obtained when continuous PSA levels and vitamin D levels were correlated. Indeed, the Spearman’s rank correlation computed to assess the relationship between PSA and vitamin D reported a negative correlation between the two variables, with r(2) = −0.123, *p* = 0.170, which did not reach statistical significance as well (Figure 2).

## 4. Discussion

Although the role of vitamin D deficiency in PCa risk has been hypothesized in several studies, the topic still remains controversial, with a lack of consistent findings in the literature. The protective vitamin D effect against PCa onset was firstly proposed, in 1990, by Schwartz and Hulka, based on the evidence that PCa risk was increased in the elderly with low serum vitamin D levels [18]. Nevertheless, more recent studies have increased the controversy on this issue. A meta-analysis involving 21 observational studies (for a total of 11,941 patients involved) performed by Xu et al. in 201 showed an elevated PCa risk in patients with increased vitamin D levels (up to 17%) [32]. Additionally, a more recent meta-analysis of 19 prospective studies, involving a total of 12,786 patients, reported a significant correlation between higher vitamin D concentration and PCa risk, suggesting per every 10 ng/mL increment of circulating vitamin D concentration, an elevation of approximately 4% of PCa risk [32,37]. A large randomized controlled trial by Manson et al., involving 25,871 participants supplemented with vitamin D or placebo for a median follow-up of 5.3 years, reported instead no differences in PCa incidence compared to placebo [35]. Another debunked hypothesis has been related to the high PCa incidence in Nordic countries due to the fluctuating sun exposure [38,39]. As reported in a Danish cohort study involving over 70,000 patients, no association was found between serum vitamin D and PCa risk, albeit overall survival was lowest for serum vitamin D deficiency [40]. A 2018 meta-analysis revealed that vitamin D supplementation not only could not be beneficial for PCa, patients but, although it was not statistically significant, it might increase the risk of overall mortality [41]. Finally, Ramakrishnan et al. showed a decreased risk of high aggressive PCa in men with an increased serum levels of vitamin D albeit the complex nature of vitamin D pathway warrants careful analysis of results obtained [42]. Several additional reports suggested an inverse relationship between vitamin D levels and the risk and aggressiveness of PCa [4,5,6,7,8,9,10,11,12,13,14,15,16,17,18,19,20,21,22,23,24,25,26,27,28,29,30,31,32,33,34,35,36,37,38,39,40,41,42,43,44,45,46]. Although there is evidence that vitamin D has tumor suppressor effects on prostatic tissue, studies on the effect of vitamin D in preventing PCa occurrence yielded inconclusive results [35,47,48].

Considering the wide variability of results obtained, it was reasonable to postulate that the effect of vitamin D levels on prostate health would be reflected also in PSA concentrations. A prospective study enrolling 105 healthy men with a physiological PSA concentration has not reported variations in PSA levels upon vitamin D administration and concomitant increase in vitamin D blood levels [16]. Another study enrolling 1705 subjects found no direct relationship between PSA and vitamin D levels in patients without PCa [33]. Furthermore, another interesting finding was reported in a meta-analysis performed by Toth et al., which showed no effects on PSA levels in different vitamin D subgroups, while in the meta-analysis performed by Shahvazi et al., PSA levels decreased in patients with vitamin D supplementation compared to placebo, although the results were not statistically significant [41,46]. Consistent with those data, our results similarly showed no association between serum vitamin D concentrations and PSA levels in healthy men. Although the number of participants enrolled was small, our findings have two major implications. Firstly, it raises concerns about the vitamin D contribution to prostate diseases associated with slightly or moderately elevated PSA levels. Secondly, it reinforces for clinicians that they should not adjust PSA reference ranges and threshold values to vitamin D levels during the decision-making process. We are conscious of different limitations of our study. First, the retrospective nature of our work, associated with the relatively limited sample size, does not permit drawing certain conclusions; secondly, the absence of a stratified PSA according to the age of patients, which, if one on side could have been an interesting aspect of our work, could have further limited the recruited sample size in smaller groups; thirdly, we assumed as a normal PSA a mean value <4 ng/mL which could not be associated with older patients [49,50,51,52,53]. We aim to evaluate these aspects and resolve these pitfalls in the next study.

## 5. Conclusions

In the present study, an absence of correlation between the serum vitamin D concentration levels and PCa risk (elevated serum PSA values) has been observed. Considering the limited sample size of the present study, further studies in a larger patient cohort and in a wider geographic area, which will also consider vitamin D supplementation, immunomarkers and other health status indicators, are needed. The role of calcium intake as a confounding factor in the vitamin D/PCa association as well as the role of solar radiation in the vitamin D metabolism should also be assessed.

## Figures and Tables

**Table 1:** Descriptive characteristics of patients enrolled.

Study Participants’ Characteristics	Mean	Standard Deviation	Median	Mode
Age	61.14	5.66	61	53
Height (cm)	174	5.26	173	178
Weight (kg)	84.92	13.15	84	90
Body Mass Index	27.97	3.53	28.03	24.22
PSA (ng/mL)	5.24	14.99	2.14	0.35
Vitamin D (ng/mL)	20.69	11.12	18.45	8.29
	**Count**		**Percentage**	
Taking drugs	29		23	
Refer a disease	26		20.6	
Hypertension	18		14.3	
Diabetes	6		4.8	
Familiar history for prostate cancer	5		4	
Smokers	7		5.6	
Alcohol	23		18.3	
Sedentary lifestyle	29		23	
Physical activity	24		19	
Altered PSA (>4 ng/mL)	50		39.7	
Vitamin D deficiency (<20.9 ng/mL)	78		61.9	
Vitamin D insufficiency (>21 ng/ml but <30 ng/mL)	31		24.6	

All the patients enrolled to the screening campaign are Caucasian men. PSA: prostate-specific antigen.

**Table 2:** Chi-square test among PSA categorical levels and vitamin D categorical levels.

			Vitamin D Levels			Total
			Deficiency	Insufficiency	Recommended	
**Abnormal PSA**	**No**	**Count**	43	22	10	75
		% within Abnormal PSA	57.3%	29.3%	13.3%	100.0%
		% within Vitamin D levels	55.1%	73.3%	58.8%	60.0%
		% of Total	34.4%	17.6%	8.0%	60.0%
	Yes	Count	35	8	7	50
		% within Abnormal PSA	70.0%	16.0%	14.0%	100.0%
		% within Vitamin D levels	44.9%	26.7%	41.2%	40.0%
		% of Total	28.0%	6.4%	5.6%	40.0%
Total		Count	78	30	17	125
		% within Abnormal PSA	62.4%	24.0%	13.6%	100.0%
		% within Vitamin D levels	100.0%	100.0%	100.0%	100.0%
		% of Total	62.4%	24.0%	13.6%	100.0%

## Data Availability

Not applicable.
